# LC-MS-Based Metabolomic Study of Oleanolic Acid-Induced Hepatotoxicity in Mice

**DOI:** 10.3389/fphar.2020.00747

**Published:** 2020-05-26

**Authors:** Hong Feng, Ying-Qiu Wu, Ya-Sha Xu, Ke-Xin Wang, Xue-Mei Qin, Yuan-Fu Lu

**Affiliations:** ^1^ Key Laboratory of Basic Pharmacology of the Ministry of Education and Joint International Research Laboratory of Ethnomedicine of the Ministry of Education, Zunyi Medical University, Zunyi, China; ^2^ Modern Research Center for Traditional Chinese Medicine, Shanxi University, Taiyuan, China

**Keywords:** oleanolic acid, metabolomics, hepatotoxicity, bile acid metabolism, LC-MS

## Abstract

Oleanolic acid (OA), a natural triterpenoid, which has the development prospects in anti-tumor therapy is a widely used hepatoprotective drug in China. It has been reported that OA can cause liver toxicity after higher doses or longer-term use. Therefore, the study aims to explore the possible hepatotoxicity mechanism based on liver metabolic profiles. Liver metabolic profiles were obtained from untargeted ultrahigh performance liquid chromatography (UHPLC)-Q Exactive Orbitrap mass spectrometry (MS) technique. It was found that altered bile acid, amino acid, and energy metabolism might be at least partly responsible for OA-induced hepatotoxicity. Bile acid metabolism, as the most important pathway, was verified by using UHPLC-TSQ-MS, indicating that conjugated bile acids were the main contributors to OA-induced liver toxicity. Our findings confirmed that increased bile acids were the key element of OA hepatotoxicity, which may open new insights for OA hepatotoxicity in-depth investigations, as well as provide a reference basis for more hepatotoxic drug mechanism research.

## Introduction

Drug-induced liver injury (DILI) is the primary factor of drug withdrawal and the incidence is increasing (Oh et al., 2015), including hepatocellular pattern, cholestatic pattern, and mixed pattern. Serious liver injury associated with Chinese herbal medicines has been reported more frequently as they become more and more popular (Oh et al., 2015). For example, the hepatotoxicity of *Polygoni Multiflori Radix* is related to activity inhibition of cytochrome P450 1A2 (CYP1A2) or cytochrome P450 2E1 (CYP2E1) (Deng et al., 2017), as well as complex composition and patient factors (Wei et al., 2019). It is a challenge in managing DILI, especially when the mechanism is unknown (Chen et al., 2015; Chatterjee and Annaert, 2018). Understanding the mechanism is essential for predicting and clinically managing drug toxicity.

As an adjuvant therapy for chronic liver disease, OA has displayed anti-tumor, anti-diabetic, and antiviral activity (Ziberna et al., 2017; Bao et al., 2020). It is frequently contained in Chinese herbal formulas as a major component of the prescription, but it has also been recorded that OA caused liver injury if given in a small dose for a long time or a large dose for a short time (Liu et al., 2019). Unfortunately, the mechanism of OA-induced hepatotoxicity has not been clarified. Thus, it is necessary to inspect the hepatotoxicity mechanism, which not only can minimize the risk of early drug development but provide diagnostic biomarkers for liver toxicity.

Metabolomics has been extensively used for investigating mechanisms at the molecular level in various research fields, e.g., drug metabolism, treatments, toxicology, etc. (Lu et al., 2018; Liu and Zhong, 2019). Moreover, metabolomics can provide valuable markers for predicting DILI (Cuykx et al., 2018). Using metabolomics, Pannala et al. identified the markers of *acetaminophen*-induced toxicity in rats, and they found that nucleotide, lipid, and amino acid metabolism were the major pathways (Pannala et al., 2019). Serum and urine metabolomics analysis from *hydrazine*-treated and control rats revealed that hydrazine altered the metabolism pathways of amino acids, glutathione metabolites, vitamins, and fatty acids (An et al., 2018). At present, liquid chromatography (LC)-mass spectrometry (MS) technology has become the mainstream tool of global metabolomics research because of superior resolution and sensitivity (Gika et al., 2014), but the results may be affected by the steps of sample collection, storage, preparation, as well as by matrix effects and the number of samples, further verification of the results through targeted analysis may be the future direction (Theodoridis et al., 2012; Chamberlain et al., 2019). Currently, few in-depth studies on key metabolic pathways have been conducted after untargeted analysis, but these in-depth mechanisms are very important for the prediction and management of drug toxicities.

In this study, in order to explore the mechanism of OA-induced hepatotoxicity and seek the diagnostic biomarkers, we employed untargeted metabolomics to analyze the metabolic differences in liver tissue after OA (457 mg/kg and 685.5 mg/kg) intragastric administration for 4 consecutive days and then verified the pivotal pathway by targeted metabolomics.

## Materials and Methods

### Materials

OA (purity >99%) was obtained from Sigma-Aldrich (Wu et al., 2018). Bile acid standards were purchased from ZZBIO Ltd. (Huang et al., 2019). HPLC grade acetonitrile and formic acid were obtained from Thermo Fisher Scientific (CA, USA), ammonium acetate was purchased from Sigma-Aldrich (St. Louis, USA).

### Animal Treatment

Male C57BL/6J mice (6–8 weeks old) were purchased from SPF Biotechnology Technology Co. Ltd. (Beijing, China). Mice were placed in a 12-h light/dark environment (8:00 a.m. to 8:00 p.m.) and fed normal chow and water ad libitum in the Key Laboratory of Basic Pharmacology of the Ministry of Education at Zunyi Medical University. After 3 days of acclimatization, the mice were randomly divided into the following three groups (n = 6), including control group, OA low-dose group (457 mg/kg, low dose), and OA high-dose group (685.5 mg/kg, high dose). The doses used were based on a previous study (Lu et al., 2013). In the control group, 10 ml/kg corn oil was given through intragastric administration once per day for 4 consecutive days. All procedures used in this study were in accordance with the requirements of the Animal Experiment Ethics Committee of Zunyi Medical University.

### Serum Biochemical Factors

Alanine aminotransferase (ALT), aspartate aminotransferase (AST), alkaline phosphatase (ALP), and total bile acids (TBA) were analyzed by standard enzymatic assays using commercial kits according to the manufacturer's protocols (Jiang-Cheng Biological, China).

### Histopathology

Liver samples were fixed in 4% neutral buffered formaldehyde solution for 24 h and embedded in paraffin. Livers were cut into 3.5 μm thick sections and the sections were stained with hematoxylin and eosin (H&E).

### Untargeted Liver Metabolic Profiling Analysis Using UHPLC- Q Exactive Orbitrap-MS

#### Liver Metabolic Sample Preparation

Liver (100 mg) was homogenized in 300 µl of ice-cold acetonitrile/water (1:1, v/v). Next, 600 µl ice-cold acetonitrile was added to 200 µl of the mixture, followed by centrifugation at 20,627 g at 4°C for 15 min. The supernatant (600 µl) was transferred to a new vial and dried in a centrifugal vacuum freeze dryer. The residue was reconstituted in 200 µl acetonitrile/water containing 0.1% formic acid. After centrifugation at 20,627 g at 4°C for 15 min, the supernatant was transferred to a sample vial for LC-MS analysis. Quality control (QC) samples were prepared by combining equal aliquots of liver, processed in the same way as the analytical samples, and were added to monitor the stability of the LC-MS platform after every eight samples (Mei, 2018).

#### Liver Metabolic Profiling Data Acquisition

Metabolic profiling of liver samples was conducted using a UHPLC-Q Exactive Orbitrap-MS (Thermo Fisher Scientific) fitted with an electrospray ionization (ESI) source. A Hypersil Gold C18 column (100×2.1 mm, 1.9 μm; Thermo Fisher Scientific) was used for metabolite separation. The column temperature was 40°C. Mobile phase A consisted of 0.1% formic acid in water and mobile phase B consisted of acetonitrile with 0.1% formic acid, the elution gradient program used was described in the previous study (Gao et al., 2018). The sample injection volume was 3 µl and the flow rate was set at 0.2 ml/min. Data were acquired using Full Scan-ddMS2 scan mode, all samples were analyzed under positive and negative ionization modes, mass parameters of the ESI ion source were set as follows: capillary temperature, 320°C; heater temperature, 300°C; sheath gas flow rate, 35 arb; auxiliary gas flow rate, 10 arb; and scan range, 100–1,500 m/z (Gong et al., 2019; Yu et al., 2019).

#### Multivariate Data Analysis (MDA)

The raw UHPLC-MS data were collected using an Xcalibur workstation (Thermo Fisher Scientific) and imported into Compound Discoverer 2.0 (Thermo Fisher Scientific) for metabolomics analysis and metabolite alignment. The processing parameters were as follows: mass range: 100–1,500 Da; mass tolerance: 5 ppm; retention time tolerance (min): 0.05; and S/N threshold: 3. After processing, the data were imported into excel to carry out MDA.

MDA was performed using SIMCA-P software (version 14.1, Umetrics, Sweden). Principal components analysis (PCA), partial least squares discriminant analysis (PLS-DA) and orthogonal partial least squares discriminant analysis (OPLS-DA) models were used to analyze the data. Variable importance in projection (VIP) > 1 indicates that a metabolite has an important influence on the group classification, and the independent sample *t*-test was used to assess the significance of the metabolites. Metabolites with VIP > 1 and *P* < 0.05 were considered statistically significant differential metabolites.

The metabolites were confirmed by using the following databases: Human Metabolome Database (http://www.hmdb.ca/), Kyoto Encyclopedia of Genes and Genomes (KEGG) LIGAND Database (https://www.genome.jp/kegg/ligand.html), and Massbank (https://massbank.eu/MassBank/Search). Significant bile acids were compared based on standard peak retention time. MetaboAnalyst (https://www.metaboanalyst.ca/) was used for metabolic pathway enrichment analysis.

### Targeted Liver Bile Acid Analysis Using UHPLC-TSQ-MS

#### Sample Preparation

The liver bile acid extraction method was described previously (Huang et al., 2019). Approximately 100 mg frozen liver was accurately weighed and homogenized in two times the volume of double-distilled deionized water (ddH_2_O; 200 µl for 100 mg). The mixture was centrifuged at 13,500 g for 15 min at 4°C, and 250 µl supernatant was transferred into a new tube. The bile acid extraction was performed with 750 µl acetonitrile and then the mixture was vortexed for 30 s and centrifuged at 13,500 g for 15 min to remove precipitated proteins and other particulates. Finally, 800 µl supernatant was transferred into a clean tube, evaporated to dryness with nitrogen, and stored at -80°C overnight. The residue was then reconstituted with 100 µl methanol/water (50:50, v/v), vortexed for 30 s, and centrifuged at 14,000 g for 10 min at 4°C.

#### Bile Acid Standard Solutions and Calibration Curves

Bile acid-free liver tissue was prepared. First, the liver homogenate was obtained by homogenizing the liver tissue in water (1:2, w/v). The liver homogenate was then treated with 150 mg/ml activated charcoal for 6 h to remove the endogenous bile acids (Huang et al., 2011; Yang et al., 2017). Twenty bile acid standards were diluted with 50% methanol/water to create final concentrations of 4–2000 ng/ml.

#### UHPLC-TSQ-MS Method

Bile acids were quantified by first conducting LC separation and then performing MS detection. We used a Hypersil Gold C18 column (100×2.1 mm, 1.9 μm, Thermo Fisher Scientific), a Finnigan Surveyor LC pump, a Finnigan Surveyor autosampler, and a TSQ Quantum triple quadrupole mass spectrometer with an ESI interface (Thermo Fisher Scientific). Mobile phase A consisted of 20% acetonitrile in water (containing 5 mM ammonium acetate), and mobile phase B consisted of acetonitrile with 20% water (containing 5 mM ammonium acetate). The flow rate was 0.3 ml/min. The gradient profile under the final LC conditions was as follows: 0–2 min, 5% B; 2–6 min, 14% B; 6.3 min, 25% B; 6.3–14 min, 50% B; 14–27 min, 95% B; 27–30 min, 100% B; and 30–34 min, 5% B. The injection volume was 3 µl. The column temperature was set at 45°C, and the sample tray temperature was maintained at 4°C. For MS detection, the ESI source was operated in the negative ion mode to produce MS/MS spectra, and Xcalibur 2.0 software was used (Thermo Fisher Scientific). High-purity nitrogen was used as the sheath gas (35 arb) and auxiliary gas (10 arb). The other parameters were as follows: spray voltage, 3.5 kV; capillary temperature, 300°C; scan width for selected reaction monitoring (SRM), 0.5 m/z; and scan time, 0.2 s. The peak width settings for both Q1 and Q3 were 0.7 m/z. The SRM ion pair transitions and collision energy levels of each component are listed in **Table 1**.

**Table 1:** Selected reaction monitoring (SRM) transitions and mass spectroscopy (MS) parameters in bile acid analysis.

Bile acid	Q1 (m/z)	Q3 (m/z)	Collision Energy (V)
TUDCA	498.3	80.0	65
UDCA	391.3	391.3	30
Nor-DCA	377.3	377.3	20
β MCA	407.3	407.3	10
CA	407.3	407.3	30
TCDCA	498.3	80.0	65
λ MCA	407.3	407.3	10
DCA	391.3	391.3	30
TLCA	482.3	80.0	60
6,7-diketoLCA	403.3	403.3	10
GCA	464.3	74.0	37
7ketoLCA	389.3	389.3	30
LCA	375.3	375.3	30
T-βMCA	514.3	80.0	60
αMCA	407.3	407.3	10
3βCA	407.3	407.3	25
CDCA	391.3	391.3	30
TCA	514.3	80.0	65
T-αMCA	514.3	80.0	60
TDCA	498.3	80.0	60

### Statistical Analysis

All results were evaluated using SPSS 18.0. All data were assessed using one-way ANOVA or Student's *t*-test. *P* < 0.05 was considered significant. The graphs were generated using GraphPad Prism version 7 (GraphPad Software). Bars represent means ± SEM.

## Results

### OA Induced Hepatotoxicity in C57BL/6J Mice

**Figure 1:**
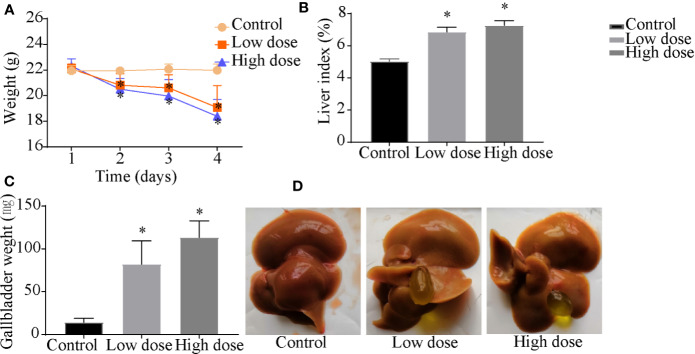
Effects of OA administration on **(A)** body weight, **(B)** liver index, **(C)** gallbladder weight, and **(D)** liver and gallbladder morphology in C57BL/6J mice. Data are expressed as mean ± SEM (n = 6). ^*^ *P* < 0.05 *vs* the control group.

**Figure 2:**
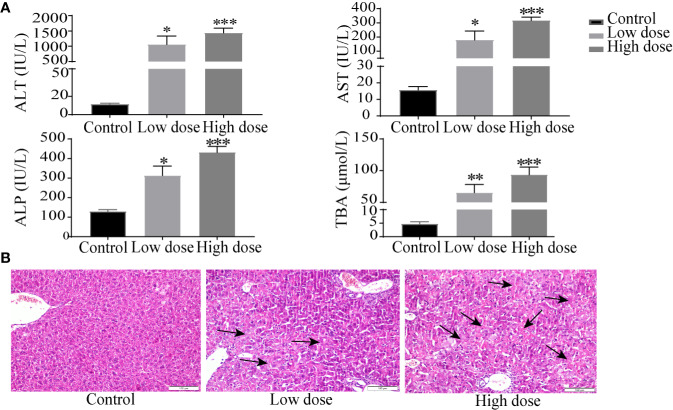
Serum biochemical factors **(A)** and liver histopathology **(B)** after OA administration in C57BL/6J mice. Data are expressed as mean ± SEM (n = 6). ^*^ *P* < 0.05, ^**^ *P* < 0.01 and ^***^ *P* < 0.001 *vs* the control group. Histological sections were stained with H&E (×200). Arrows represent hepatocellular cell death.

Compared with the control group, the weight of the mice decreased significantly in low- and high-dose groups (*P* < 0.05, **Figure 1A**), and there was no significant difference between low dose and high dose. The liver index and gallbladder weight increased (*P* < 0.05, **Figures 1B, C**), lighter liver color, and gallbladder enlargement were observed after OA administration groups (**Figure 1D**), but without any statistical significance between low dose and high dose. The levels of ALT, AST, ALP, TBA increased by 95-, 10-, 1.4-, 12-fold in the low-dose group and by 129-, 20-, 2.3-, 18-fold in the high-dose group compared with the control group (*P* < 0.05, **Figure 2A**), respectively. The liver pathology was agreed with the serum biochemical results, hepatocyte necrosis (black arrows) were evident in both low- and high-dose groups (**Figure 2B**). The results suggested that OA-mediated liver injury was mainly associated with the changes of bile acids.

### OA Altered Liver Metabolic Profile

**Figure 3:**
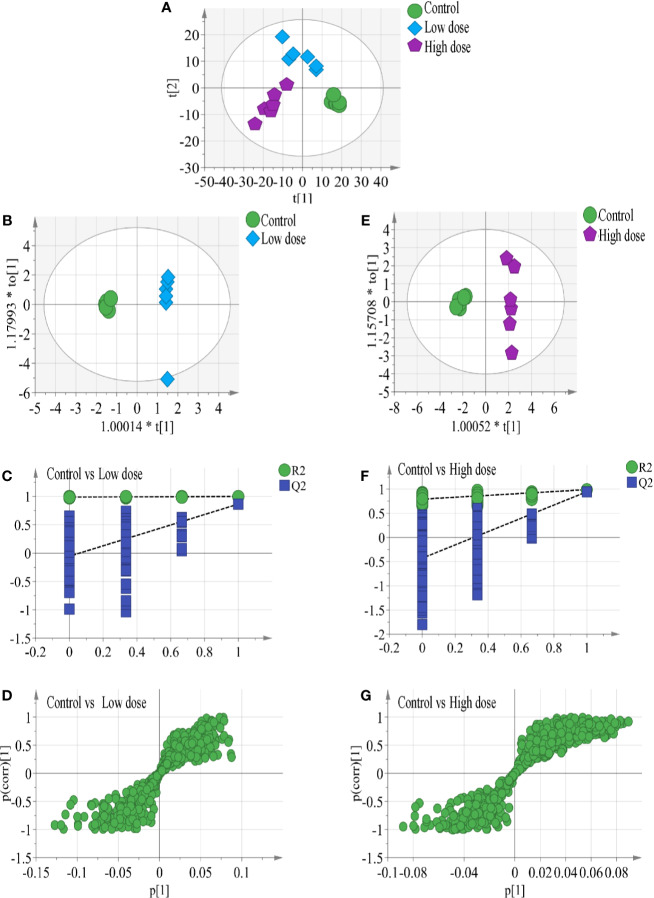
**(A)** Results of multiple pattern recognition methods regarding liver metabolites after OA administration in C57BL/6J mice, showing PLS-DA score plot (n = 6, R^2^Y = 0.99, R^2^X = 0.664, Q^2^ 543 = 0.878). OPLS score plot of **(B)** low-dose group (n = 6, R^2^Y = 0.969, R^2^X = 0.663, Q^2^ = 0.749) and **(E)** high-dose group (n = 6, R^2^Y = 0.988, R^2^X = 0.634, Q^2^ = 0.945) with the control group. Permutation test results of **(C)** low-dose group (R^2^ = 0.99, Q^2^ = −0.0654) and **(F)** high-dose group (R^2^ = 0.779, Q^2^ = −0.353) with the control group. OPLS S-plot of **(D)** low-dose group and **(G)** high-dose group with the control group. Each dot in the S-plot represents an ion. Ions far away from the origin are potential biomarkers.

In the work, the PCA score plot of all samples, including QC samples, was applied to demonstrate the LC-MS system stability (**Figure S1**), QC samples were clustered close to the center. The PLS-DA statistical method was employed to assess the metabolic changes among all groups. The cumulative R^2^Y and Q^2^ of PLS-DA model indicated good prediction and reliability (**Figure 3A**), hinting that endogenous metabolites changed in the two administration groups and in the control group.

To completely distinguish metabolites in the OA groups from those in the control group and identify potential biomarkers, we used OPLS-DA (**Figures 3B, E**) to eliminate irrelevant spectral changes. The R^2^Y and Q^2^ parameters represent the reliability of the multiple pattern recognition methods. R^2^Y represents how well the model fits the data, and Q^2^ indicates the predictive accuracy of the model. The results of the permutation tests (200 permutations) showed that the two models were not overfitted and reflected the metabolic changes (intercepts: R^2^ = 0.99, Q^2^ = −0.0654, and R^2^ = 0.779, Q^2^ = −0.353) (**Figures 3C, F**). The corresponding OPLS S-plots (**Figures 3D, G**) in turn showed the contributions of different variables. Ions far away from the origin might be potential biomarkers. Finally, eleven significantly differential metabolites were identified between the low-dose group and the control group, fifteen significantly differential metabolites were identified between the high-dose group and the control group. All metabolite information was listed in **Table 2**.

**Table 2:** Identified differential metabolites in the liver of C57BL/6J mice with or without OA administration.

NO.	Name	RT (min)	Formula	M/Z	Relative content			Ion	Metabolic pathway
					control	low-dose	high-dose		
1	Valine	2.69	C_5_H_11_NO_2_	118.0862	0.0724 ± 0.0041	0.1355 ± 0.0051	/	+	Valine, leucine and isoleucine degradation; Pantothenate and CoA biosynthesis
2	D-Mannose	2.11	C_6_H_12_O_6_	179.0559	0.1552 ± 0.0073	/	0.0263 ± 0.0010	–	Biosynthesis of alkaloids derived from ornithine, lysine, and nicotinic acid
3	Pantothenic acid	6.65	C_9_H_17_NO_5_	220.1177/218.1031	0.0388 ± 0.0030	/	0.2724 ± 0.0132	+/-	Beta-Alanine metabolism; Pantothenate and CoA biosynthesis
4	Tryptophan	6.77	C_11_H_12_N_2_O_2_	205.0969	0.0585 ± 0.0039	0.1258 ± 0.0010	/	+	Tryptophan metabolism
5	Taurochenodeoxycholic acid	11.93	C_26_H_45_NO_6_S	498.29	0.0097 ± 0.0026	0.0937 ± 0.0084	0.1080 ± 0.0039	–	Primary bile acid biosynthesis; Bile secretion
6	Uracil	3.44	C_4_H_4_N_2_O_2_	113.0346	0.0552 ± 0.0043	/	0.1753 ± 0.0119	+	Pyrimidine metabolism; Beta-Alanine metabolism; Pantothenate and CoA biosynthesis
7	Allantoin	2.21	C_4_H_6_N_4_O_3_	157.0365	0.00381 ± 0.0022	/	0.3413 ± 0.0280	–	Purine metabolism
8	Taurodeoxycholic acid	12.88	C_26_H_45_NO_6_S	498.2896/500.3037	0.1897 ± 0.0110	0.0472 ± 0.0135	0.0187 ± 0.0038	+/-	Primary bile acid biosynthesis; Bile secretion
9	4-Hydroxybutyric acid	5.11	C_4_H_8_O_3_	103.04	0.0631 ± 0.0056	/	0.1705 ± 0.074	–	Butanoate metabolism
10	2-Hydroxyvaleric acid	7.09	C_5_H_10_O_3_	117.0557	0.0331 ± 0.0019	/	0.2376 ± 0.0152	–	Other
11	Glycocholic acid	10.73	C_26_H_43_NO_6_	466.3154/464.3015	0.0079 ± 0.0009	0.0772 ± 0.0102	0.1017 ± 0.0149	+/-	Primary bile acid biosynthesis; Bile secretion
12	Suberic acid	7.49	C_8_H_14_O_4_	173.0817	0.0972 ± 0.0052	/	0.2324 ± 0.0159	–	Other
13	4′-Phosphopantetheine	6.63	C_11_H_23_N_2_O_7_PS	357.0891	0.0332 ± 0.0037	0.1563 ± 0.0148	0.1500 ± 0.0147	–	Biosynthesis of antibiotics
14	Citraconic acid	6.61	C_5_H_6_O_4_	129.0192	0.0148 ± 0.0018	0.1159 ± 0.0183	0.2713 ± 0.0206	–	Arginine and proline metabolism
15	Succinate	3.60	C_4_H_4_O_4_	115.0037	0.0597 ± 0.0019	0.1395 ± 0.0056	0.1205 ± 0.0116	–	Biosynthesis of alkaloids derived from ornithine, lysine, and nicotinic acid
16	Deoxycholic acid	16.66	C_24_H_40_O_4_	391.2855	0.0684 ± 0.0063	0.1540 ± 0.0111	/	–	Primary bile acid biosynthesis; Bile secretion
17	L-Glutamic acid	2.58	C_5_H_9_NO_4_	148.0604	0.2123 ± 0.0249	0.0535 ± 0.0103	0.0718 ± 0.0134	+	Arginine and proline metabolism
18	Citric acid	2.81	C_6_H_8_O_7_	191.0195	0.0466 ± 0.0020	0.1351 ± 0.0083	0.1331 ± 0.0064	–	Biosynthesis of amino acids

### Characterization of Potential Biomarkers

**Figure 4:**
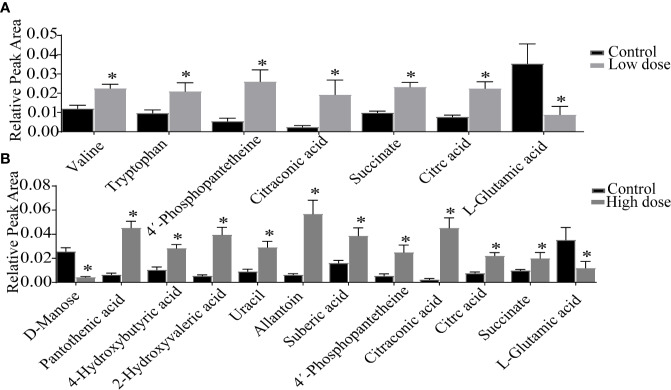
Detailed relative changes in the relative peak area of potential biomarkers. **(A)** Control group *vs* low-dose group, **(B)** Control group *vs* high-dose group. Data are expressed as mean ± SEM (n = 6). *P < 0.05 *vs* the control group.

Detailed relative changes in potential biomarkers were shown in **Figure 4** and **Figure S2**. Compared with the control group, taurodeoxycholic acid (TDCA) and L-glutamic acid were downregulated in both low- and high-dose groups (*P* < 0.05). Valine, tryptophan, taurochenodeoxycholic acid (TCDCA), glycocholic acid (GCA), 4′-phosphopantetheine, citric acid, and succinate were upregulated in the low-dose group (*P* < 0.05). Compared with the control group, pantothenic acid, 4-Hydroxybutyric acid, 2-Hydroxyvaleric acid, uracil, allantoin, 4′-phosphopantetheine, citric acid, succinate, citraconic acid, and TCDCA were upregulated in the high-dose group (*P* < 0.05). These differential metabolites could be used to effectively distinguish the groups.

### Metabolic Pathway Analysis

**Figure 5:**
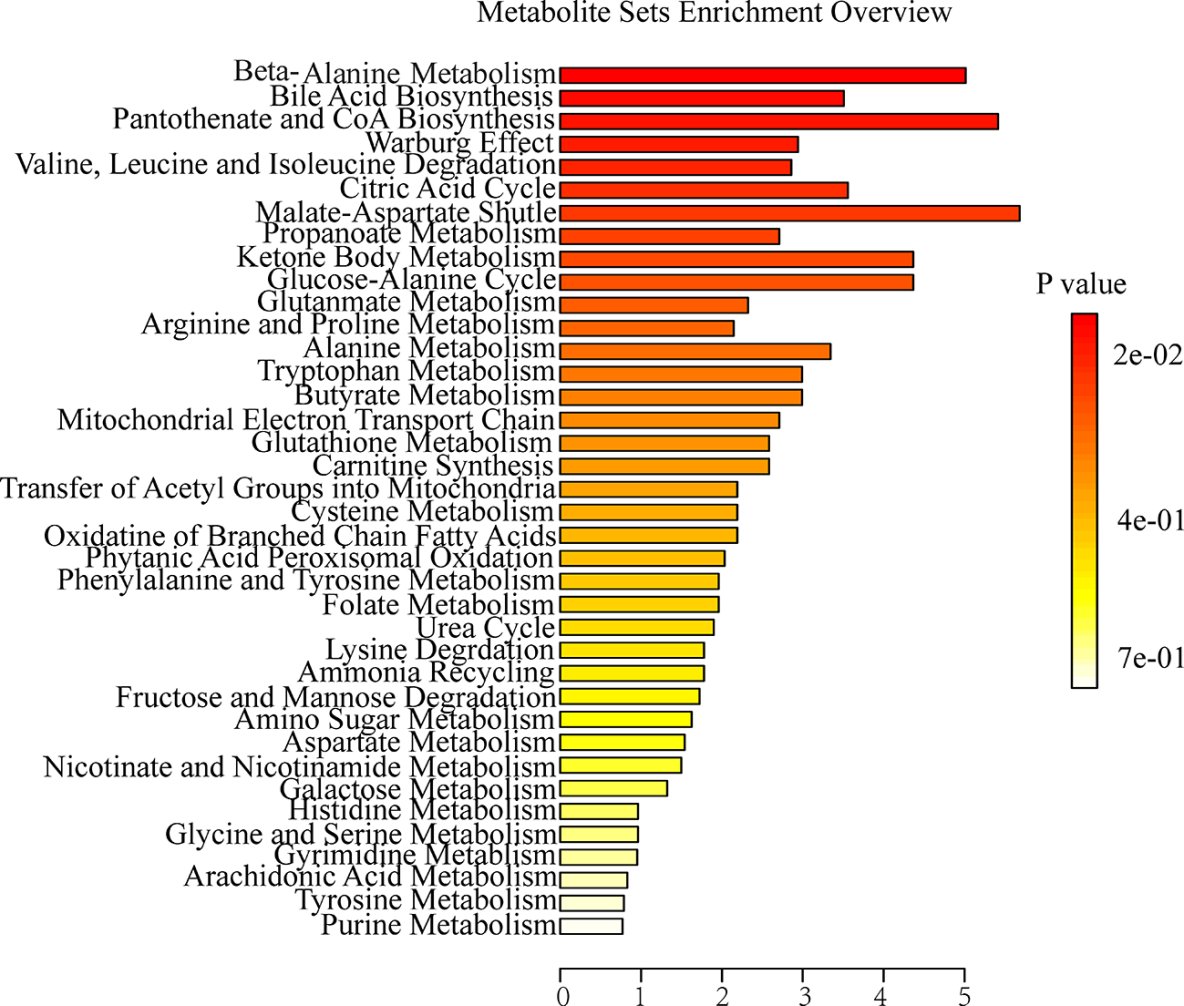
Summary of metabolite set enrichment after OA administration in C57BL/6J mice using MetaboAnalyst.

To further explore the association between the markedly changed metabolites in all OA administration groups and OA hepatotoxicity. MetaboAnalyst was used to reveal the possible metabolic pathways, based on the *P* values (*P* < 0.05). Among them, amino acid metabolism (beta-alanine metabolism; valine, leucine and isoleucine degradation), energy metabolism (citric acid cycle; pantothenate and CoA biosynthesis), and bile acid metabolism (bile acid biosynthesis) were considered as the pertinent metabolic pathways (**Figure 5**).

### Correlation of Serum Biochemical and Metabolites

**Figure 6:**
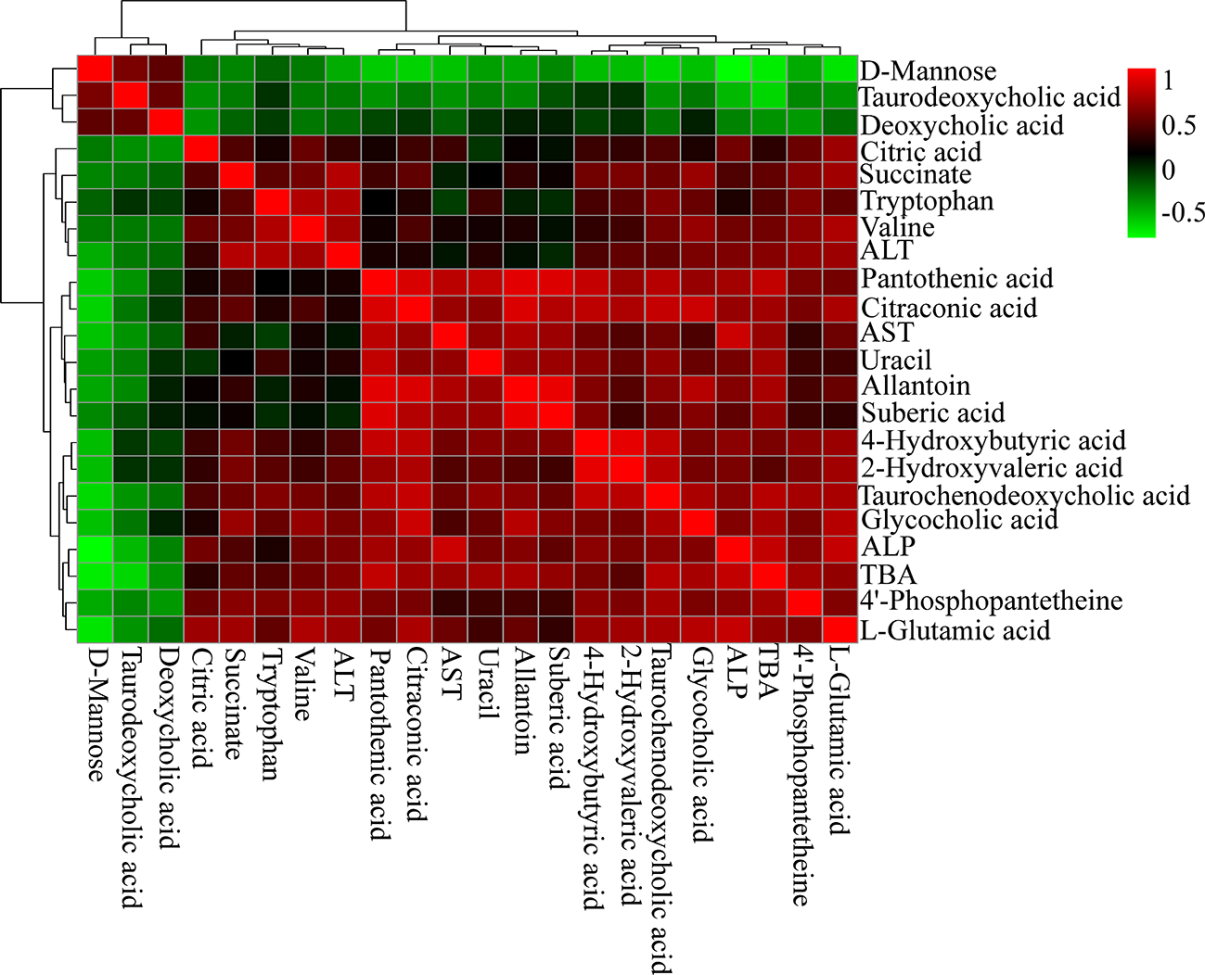
Correlation analysis results of OA-induced liver marker metabolites and biochemical factors (ALT, AST, ALP, and TBA). Red squares indicate a positive correlation, and green squares indicate a negative correlation.

Spearman's correlation analysis was performed to explore the relationships between differential metabolites and serum biochemical factors ALT, AST, ALP, TBA (**Figure 6**). There were strong positive correlations among TCDCA, GCA, 4-Hydroxybutyric acid, 2-Hydroxyvaleric acid, and 4′-phosphopantetheine, and strong negative correlations among deoxycholic acid (DCA), TDCA, and D-Mannose. The observation of both positive and negative correlations between bile acids and serum biochemical factors suggested that OA-induced hepatotoxicity was closely correlated with bile acid homeostasis.

### Analysis of Bile Acids Using UHPLC-TSQ-MS/MS

**Figure 7:**
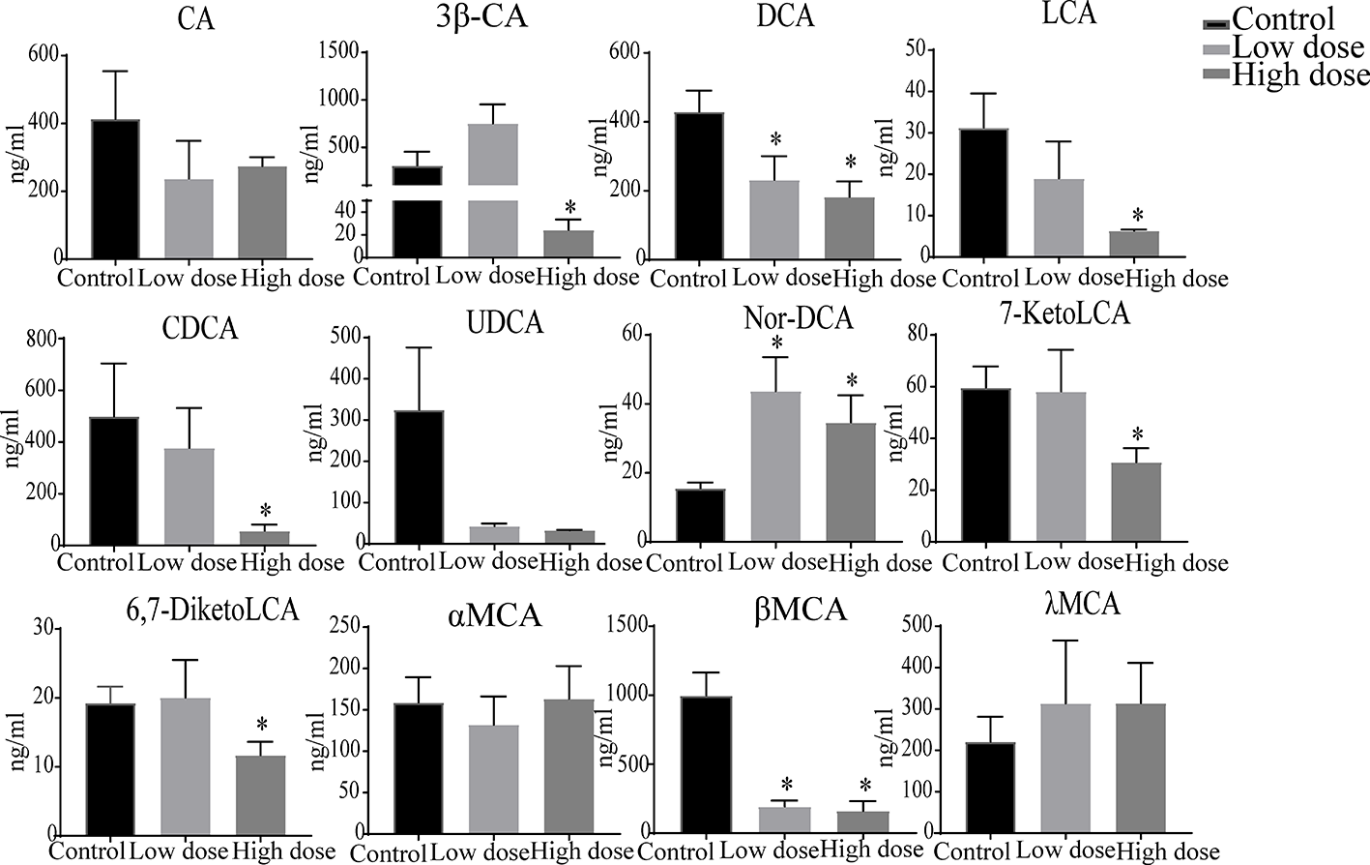
Levels of unconjugated bile acids in liver samples after OA administration in C57BL/6J mice. Data are expressed as mean ± SEM (n = 6). ^*^ *P* < 0.05 *vs* the control group.

**Figure 8:**
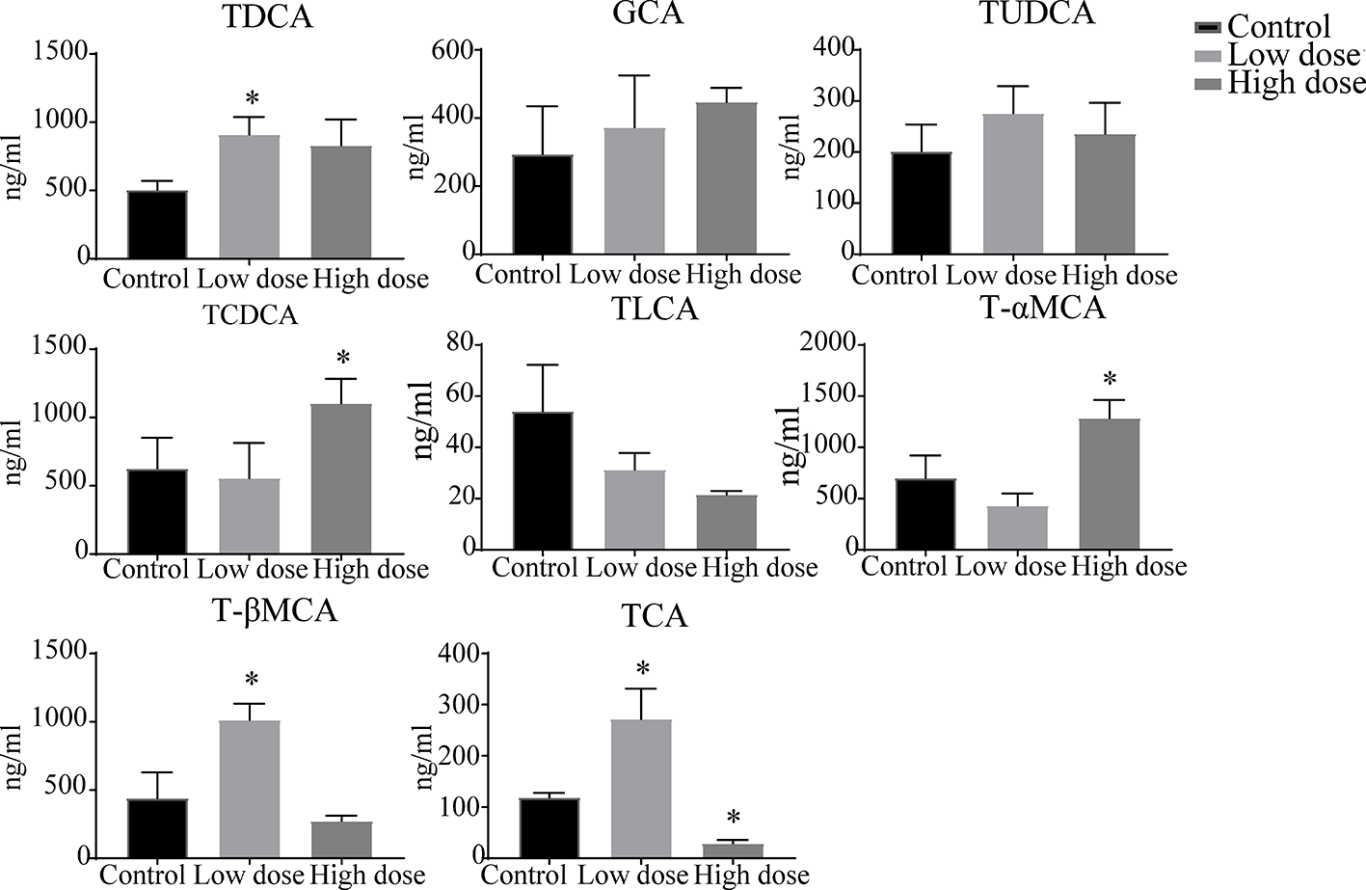
Levels of conjugated bile acids in liver samples after OA administration in C57BL/6J mice. Data are expressed as mean ± SEM (n = 6). ^*^ *P* < 0.05 *vs* the control group.

The liver levels of both unconjugated and conjugated bile acids were changed by OA (**Figures 7** and **8**). With respect to the unconjugated bile acids, the concentrations of ursodeoxycholic acid (UDCA), lithocholic acid (LCA), DCA, beta-muricholic acid (βMCA), 3β-cholic acid (3β-CA), 7-ketholithocholic acid (7-ketoLCA), and 6,7-diketolithocholic acid (6,7-diketoLCA) were decreased, but 23-nordeoxycholic acid (Nor-DCA) were increased after OA administration compared with the control group. In regard to the conjugated bile acids, the concentrations of TCDCA, tauro-alpha-muricholic acid sodium salt (T-αMCA), tauro-beta-muricholic acid sodium salt (T-βMCA), and taurocholic acid (TCA) were increased (*P* < 0.05), taurolithocholic acid (TLCA) was decreased (*P* < 0.05) after OA administration compared with that of corresponding values in the control group. Collectively, bile acid changes were strongly related to OA-induced hepatotoxicity.

**Figure 9:**
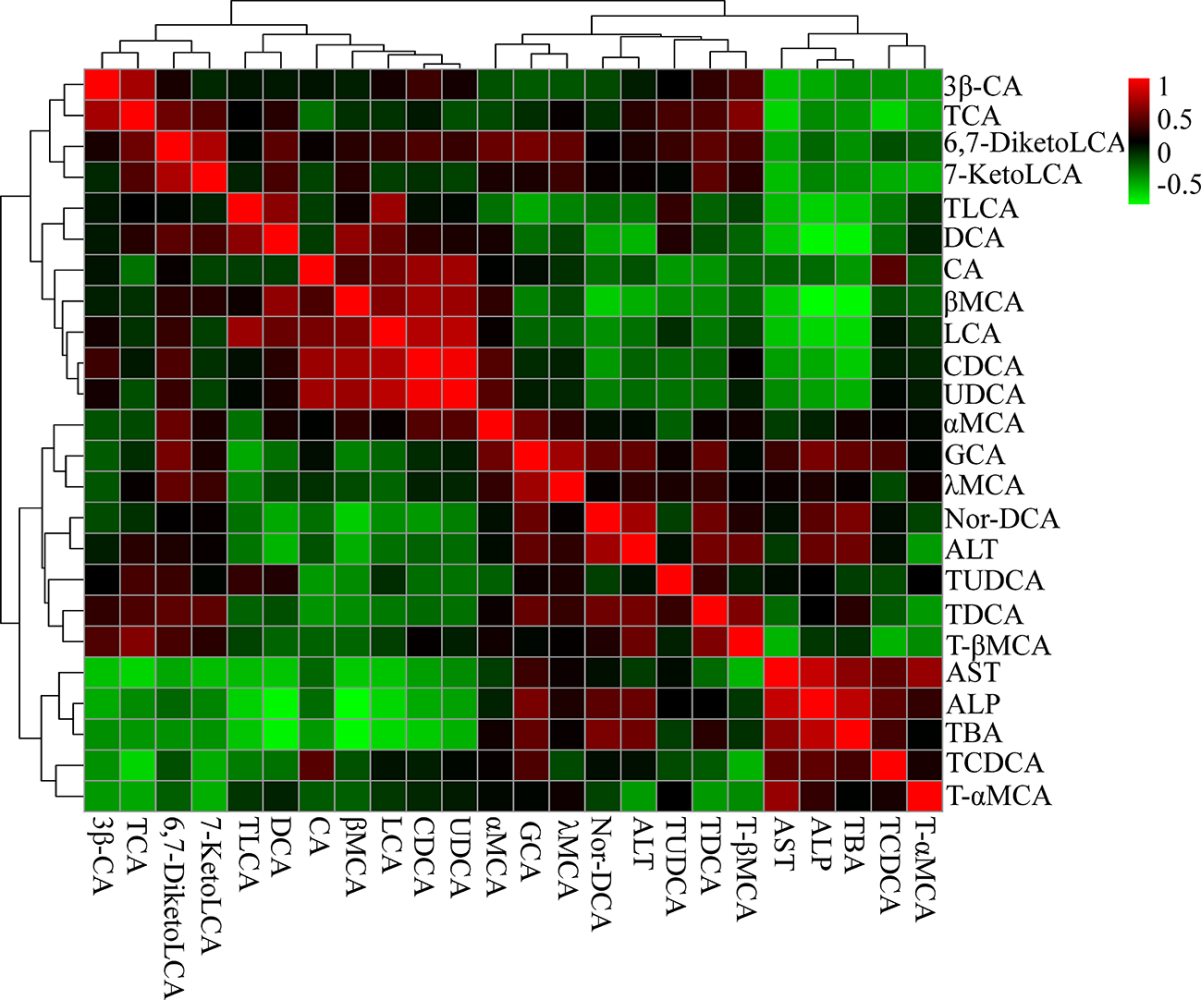
Correlation analysis results of liver bile acids and biochemical factors (ALT, AST, ALP, and TBA). Red squares indicate a positive correlation, and green squares indicate a negative correlation.

We then examined the correlations between bile acids and serum biochemical factors (**Figure 9**). The results were in accordance with the metabolomics analysis. ALT was positively correlated with GCA, TDCA, and T-βMCA, and negatively correlated with UDCA, chenodeoxycholic acid (CDCA), LCA, CA, DCA, and TLCA. ALP and TBA were positively correlated with TCDCA, GCA, and Nor-DCA, and negatively correlated with UDCA, CDCA, LCA, DCA, and TLCA.

## Discussion

In this experiment, we observed that OA caused liver damage after given different doses, produced a dose-dependent increase in serum biochemical factors. To define the mechanism involved in OA hepatotoxicity. We firstly detected endogenous substances using the untargeted metabolomics approach and found that OA interfered bile acid, energy, and amino acid metabolism. Combined with the endogenous substances, biochemical analysis, and gallbladder morphology, bile acid components were then investigated by targeted metabolomics, the results unmasked that increased bile acids were the main factor leading to OA hepatotoxicity.

The liver plays a crucial role in amino acid metabolism, it is responsible for a large part of the overall amino acid synthesis and catabolism. The alteration of amino acid metabolism has recently is related to liver injury (Zhao et al., 2017), and increased or decreased amino acids were as potential hepatotoxicity biomarkers (An et al., 2020). Similarly, compared with the control group, L-glutamic acid was decreased both in low- and high-dose groups (**Figure 4**), suggesting that OA disturbed amino acid metabolism. Notably, other research suggested that valine and tryptophan were increased in animal models of liver injury (Osawa et al., 2011; Chang et al., 2017), we found that valine and tryptophan increased in low dose but not significantly changed in high dose, which suggested that amino acid metabolism disorder caused by valine and tryptophan was not the main reason of OA liver toxicity.

The TCA cycle is an important hub related to carbohydrate, fat, and protein metabolism, of which impairments are closely associated with liver injury, impacting energy metabolism (Sunny et al., 2011). Consistent with observations in *CCl4*-induced acute liver injury (Zhang et al., 2018), elevated citraconic acid, succinate, and citric acid were observed in low- and high-dose groups (**Figure 4**). According to the report (Zhao et al., 2018), 4′-phosphopantetheine was a key intermediate in the production of CoA, which was positively correlated with ALT and AST. The increased level of 4′-phosphopantetheine also showed that OA destroyed energy metabolism. Overall, destroyed energy metabolism would potentially contribute to OA liver injury.

Apart from altered amino acid metabolism and energy metabolism, bile acid metabolism was similarly perturbed pathways after OA administration. Bile acids are synthesized from cholesterol in the liver, of which change can directly affect the metabolic state of the liver and indicate the degree of liver injury (Song et al., 2011), the increased bile acids are considered as diagnostic markers of drug-induced cholestatic liver injury (Beuers et al., 2015; Luo et al., 2018). The finding that levels of bile acids TCDCA, GCA were increased and TDCA was decreased in both low- and high-dose groups (**Figure S2**), DCA was increased in low dose group, we noted that TCDCA, GCA was positive with serum biochemical factors, while TDCA and DCA were negatively correlated with them. Although potential changes of bile acid composition in OA management mice have not been determined, it is attractive to speculate that bile acid homeostasis imbalance results in OA hepatotoxicity. In addition, elevated TBA, ALP as well as gallbladder morphology after OA administration (**Figures 1** and **2A**) may mostly reflect the bile acid homeostasis imbalance. Since it may be hypothesized that bile acid metabolism is the key metabolism pathway underlying OA-induced hepatotoxicity, the targeted metabolomics method was then implemented to further verify bile acid metabolism alterations.

Drugs caused cholestatic liver injury by changing the relative composition or concentration of the bile acids (Chatterjee and Annaert, 2018), characteristic as bile duct hyperplasia, necrosis, inflammatory cells, feathery degeneration, and steatosis. In terms of our observations, hepatocyte necrosis happened in low- and high-dose groups, but liver necrosis was more severe in the high dose group (**Figure 2B**). Importantly, studies noted that increased bile acids such as CA, α/βMCA, GCA, TCDCA, TCA, and T-α/βMCA were regarded as diagnostic markers of cholestatic liver damage in the liver or serum (Luo et al., 2013; Tang et al., 2016; Tian et al., 2017). In our study, we found that the large proportion of conjugated bile acids (TDCA, TCDCA, TUDCA, GCA, and T-βMCA) were increased after OA administration (**Figure 8**), and the overall content of unconjugated bile acids were decreased (**Figure 7**), indicating that conjugated bile acids were the main contributors of OA hepatotoxicity, and while providing a reference for finding diagnostic indicators of OA hepatotoxicity. Moreover, it is known that cholestasis is caused by the imbalance of bile acids regulation, and Farnesoid X receptor (FXR) plays an important role in bile acids regulation, inhibition of FXR can augment the accumulation of bile acids in the liver, and subsequently cause liver injury (Guo et al., 2016; Shin and Wang, 2019), T-βMCA is a natural antagonist of FXR (Takahashi et al., 2016), the correlation analysis showed that T-βMCA were positively correlated with ALT (**Figure 9**), implying that suppressed FXR was a key factor in OA hepatotoxicity, but further study is needed to confirm. Unexpectedly, conjugated bile acids TCA and T-βMCA only increased in the low-dose group and appeared to be decreased in the high-dose group (**Figure 8**), possibly due to these bile acids that increased in serum or other tissues in the high dose group (Woolbright et al., 2014).

Surprisingly, unconjugated bile acids such as CA, DCA, CDCA, and LCA (**Figure 9**) were negatively correlated with the ALT, TBA, and ALP, which was inconsistent with literature reports (Tang et al., 2016), but could be explained by unconjugated bile acids having little influence on OA hepatotoxicity. Furthermore, 3β-CA was increased in the low-dose group, decreased in the high-dose group, probably be considered as a defense mechanism liver injury caused by conjugated bile acids (Li et al., 2017). In all, the changes of unconjugated bile acids further proved that the hepatotoxicity of OA was mainly caused by the accumulation of conjugated bile acids in the liver. Interestingly, in **Figure 7**, DCA as well as other unconjugated bile acids changed in the high dose but not in **Figure S2**, and GCA was no significantly changed that was not similar to the **Figure S2**, the phenomenon may be explained by the results of matrix effect and the limited sample size in untargeted metabolomics analysis, which may have reduced the statistical power (Wu et al., 2017; Wang et al., 2018). Future studies can advance our findings by enlarging sample size or exploring the metabolites of serum and other tissues.

In conclusion, the above results demonstrated that OA destroyed energy, amino acid and bile acid metabolism. However, we have shown that bile acid metabolism is likely to be the most important pathway involved in the OA-induced liver injury (**Figure S3**). The bile acids (especially conjugated bile acids such as T-βMCA, TCA, and TCDCA) can be as the potential biomarkers, which may be helpful for further investigations of OA hepatotoxicity mechanism, as well as provide a reference basis for more studies on hepatotoxic drug mechanism.

## Data Availability Statement

All datasets generated for this study are included in the article/**Supplementary Material**.

## Ethics Statement

The animal study was reviewed and approved by Animal Experiment Ethics Committee of Zunyi Medical University.

## Author Contributions

Y-FL, Y-SX, and HF designed the research. HF performed the main experiments and wrote the paper. Y-FL revised the paper. Y-QW participated in part of the experiments. K-XW and X-MQ directed the UHPLC-MS data analysis. All authors approved the final version of the manuscript.

## Funding 

This work was funded by the Natural Science Foundation of China (Grant No. 81760678 and 81460632); the 2011 Collaborative Innovation Center of Guizhou Traditional Chinese Medicine and Ethnic Medicine (Grant No. Qianjiaokeyanfa [2012] 311); and the First-Class Disciplines Fund of the Education Department of Guizhou Province (Grant No. GNYL [2017] 006 YLXKJS-YS-05).

## Conflict of Interest

The authors declare that the research was conducted in the absence of any commercial or financial relationships that could be construed as a potential conflict of interest.

## Supplementary Material

Figure S1 PCA score plots of C57BL/6J mice liver from different groups (n = 6, R^2^X = 0.593, Q^2^ 543 = 0.217).

Figure S2 Detailed relative changes in the relative peak area of potential biomarkers. **(A)** Control group *vs* low-dose group, **(B)** Control group *vs* high-dose group. Data are expressed as mean ± SEM (n = 6). **P* < 0.05 vs the control group.

Figure S3 Schematic diagram of OA hepatotoxicity.

## Abbreviations

TUDCA, tauroursodeoxycholic acid; CDCA, chenodeoxycholic acid; CA, cholic acid; UDCA, ursodeoxycholic acid; DCA, deoxycholic acid; TCA, taurocholic acid; TCDCA, taurochenodeoxycholic acid; TDCA, taurodeoxycholic acid; αMCA, αmuricholic acid; βMCA, βmuricholic acid; TLCA, taurolithocholic acid; GCA, glycocholic acid; LCA, lithocholic acid; 6,7-diketoLCA, 6,7-diketolithocholic acid; 7-ketoLCA, 7-ketholithocholic acid; nor-DCA, 23-nordeoxycholic acid; λMCA, gamma muricholic acid; T-αMCA, tauro-alpha-muricholic acid sodium salt; T-βMCA, tauro-beta-muricholic acid sodium salt.

## References

[B1] An Z. Li C. Lv Y. Li P. Wu C. Liu L. (2018). Metabolomics of hydrazine-induced hepatotoxicity in rats for discovering potential biomarkers. Dis. Markers. 2018, 8473161. 10.1155/2018/8473161 29849827 PMC5914126

[B2] An Z. Hu T. Lv Y. Li P. F. Liu L. H. (2020). Targeted amino acid and related amines analysis based on iTRAQ^®^-LC-MS/MS for discovering potential hepatotoxicity biomarkers. J. Pharm. Biomed. Anal. 178, 112812. 10.1016/j.jpba.2019.112812 31639596

[B3] Bao Y. Zhang S. Chen Z. Chen A. T. Ma J. Deng G. (2020). Synergistic chemotherapy for breast cancer and breast cancer brain metastases via Paclitaxel-loaded Oleanolic Acid nanoparticles. Mol. Pharm. 17, 1343–1351. 10.1021/acs.molpharmaceut.0c00044 32150416

[B4] Beuers U. Trauner M. Jansen P. Poupon R. (2015). New paradigms in the treatment of hepatic cholestasis: from UDCA to FXR, PXR and beyond. J. Hepatol. 62 (1 Suppl), S25–S37. 10.1016/j.jhep.2015.02.023 25920087

[B5] Chamberlain C. A. Rubio V. Y. Garrett T. J. (2019). Impact of matrix effects and ionization efficiency in non-quantitative untargeted metabolomics. Metabolomics. 15, 135. 10.1007/s11306-019-1597-z 31584114

[B6] Chang H. Meng H. Y. Liu S. M. Wang Y. Yang X. X. Lu F. (2017). Identification of key metabolic changes during liver fibrosis progression in rats using a urine and serum metabolomics approach. Sci. Rep. 7, 11433. 10.1038/s41598-017-11759-z 28900168 PMC5595818

[B7] Chatterjee S. Annaert P. (2018). Drug-induced cholestasis: mechanisms, models, and markers. Curr. Drug Metab. 19, 808–818. 10.2174/1389200219666180427165035 29708070

[B8] Chen M. Suzuki A. Borlak J. Andrade R. J. Lucena M. I. (2015). Drug-induced liver injury: interactions between drug properties and host factors. J. Hepatol. 63, 503–514. 10.1016/j.jhep.2015.04.016 25912521

[B9] Cuykx M. Rodrigues R. M. Laukens K. Vanhaecke T. Covaci A. (2018). In vitro assessment of hepatotoxicity by metabolomics: a review. Arch. Toxicol. 92, 3007–3029. 10.1007/s00204-018-2286-9 30155722

[B10] Deng K. L. Jing C. Zhen Z. G. Sun Z. X. (2017). Hepatotoxicity in rats induced by aqueous extract of Polygoni Multiflori Radix, root of Polygonum multiflorum related to the activity inhibition of CYP1A2 or CYP2E1. Evid. Based. Complement. Alternat. Med. 2017, 9456785. 10.1155/2017/9456785 28626488 PMC5463189

[B11] Gao X. Liang M. Fang Y. Zhao F. Tian J. Zhang X. (2018). Deciphering the differential effective and toxic responses of Bupleuri Radix following the induction of chronic unpredictable mild stress and in healthy rats Based on Serum Metabolic Profiles. Front. Pharmacol. 8, 995. 10.3389/fphar.2017.00995 29379441 PMC5775221

[B12] Gika H. G. Theodoridis G. A. Plumb R. S. Wilson I. D. (2014). Current practice of liquid chromatography-mass spectrometry in metabolomics and metabolomics. J. Pharm. Biomed. Anal. 87, 12–25. 10.1016/j.jpba.2013.06.032 23916607

[B13] Gong W. Zhu S. Chen C. Yin Q. Li X. Du G. (2019). The anti-depression effect of angelicae sinensis radix is related to the pharmacological activity of modulating the hematological anomalies. Front. Pharmacol. 10, 192. 10.3389/fphar.2019.00192 30894817 PMC6414447

[B14] Guo H. L. Hassan H. M. Zhang Y. Dong S. Z. Ding P. P. Wang T. (2016). Pyrazinamide induced rat cholestatic liver injury through inhibition of FXR regulatory effect on bile acid synthesis and transport. Toxicol. Sci. 152, 417–428. 10.1093/toxsci/kfw098 27255380

[B15] Huang J. Bathena S. P. R. Iván L. Alnouti Y. (2011). Simultaneous characterization of bile acids and their sulfate metabolites in mouse liver, plasma, bile, and urine using LC-MS/MS. J. Pharm. Biomed. Anal. 55, 1111–11119. 10.1016/j.jpba.2011.03.035 21530128

[B16] Huang S. Wu Q. Liu H. Ling H. He Y. Wang C. (2019). Alkaloids of Dendrobium nobile Lindl. altered hepatic lipid homeostasis via regulation of bile acids. J. Ethnopharmacol. 241, 111976. 10.1016/j.jep.2019.111976 31132462

[B17] Li Y. Tang R. Leung P. S. C. Gershwin M. E. Ma X. (2017). Bile acids and intestinal microbiota in autoimmune cholestatic liver diseases. Autoimmun. Rev. 16, 885–896. 10.1016/j.autrev.2017.07.002 28698093

[B18] Liu Q. T. Zhong X. Y. (2019). Application of metabolomics in neonatal clinical practice. Zhongguo Dang Dai Er Ke Za Zhi. 21, 942–948. 10.7499/j.issn.1008-8830.2019.09.019 31506158 PMC7390243

[B19] Liu J. Lu Y. F. Wu Q. Xu S. F. Shi F. G. Klaassen C. D. (2019). Oleanolic acid reprograms the liver to protect against hepatotoxicants, but is hepatotoxic at high doses. Liver Int. 39, 427–439. 10.1111/liv.13940 30079536

[B20] Lu Y. F. Wan X. L. Xu Y. Liu J. (2013). Repeated oral administration of oleanolic acid produces cholestatic liver injury in mice. Molecules 18, 3060–3071. 10.3390/molecules18033060 23470335 PMC6270117

[B21] Lu Y. Zhao X. M. Hu Z. Wang L. Li F. (2018). LC-MS-based metabolomics in the study of drug-induced liver injury. Curr. Pharmacol. Rep. 5, 56–67. 10.1007/s40495-018-0144-3

[B22] Luo L. Schomaker S. Houle C. Aubrecht J. Colangelo J. L. (2013). Evaluation of serum bile acid profiles as biomarkers of liver injury in rodents. Toxicol. Sci. 137, 12–25. 10.1093/toxsci/kft221 24085190

[B23] Luo L. Aubrecht J. Li D. Warner R. L. Johnson K. J. Kenny J. (2018). Assessment of serum bile acid profiles as biomarkers of liver injury and liver disease in humans. PloS One 13, e0193824. 10.1371/journal.pone.0193824 29513725 PMC5841799

[B24] Mei L. L. (2018). Study on the relationship of the anti-depression/toxicity and the dosage of petroleum ether fraction of bupleuri radix based on LC-MS metabolomics. [master's thesis]. (Taiyuan: Shanxi University).

[B25] Oh S. J. Cho J. H. Son C. G. (2015). Systematic review of the incidence of herbal drug-induced liver injury in Korea. J. Ethnopharmacol. 159, 253–256. 10.1016/j.jep.2014.11.027 25460587

[B26] Osawa Y. Kanamori H. Seki E. Hoshi M. Ohtaki H. Yasuda Y. (2011). L-tryptophan-mediated enhancement of susceptibility to nonalcoholic fatty liver disease is dependent on the mammalian target of rapamycin. J. Biol. Chem. 286, 34800–34808. 10.1074/jbc.M111.235473 21841000 PMC3186417

[B27] Pannala V. R. Vinnakota K. C. Rawls K. D. Estes S. K. O'Brien T. P. Printz R. L. (2019). Mechanistic identification of biofluid metabolite changes as markers of acetaminophen-induced liver toxicity in rats. Toxicol. Appl. Pharmacol. 372, 19–32. 10.1016/j.taap.2019.04.001 30974156 PMC6599641

[B28] Shin D. J. Wang L. (2019). Bile acid-activated aeceptors: a review on FXR and other nuclear receptors. Handb. Exp. Pharmacol. 256, 51–72. 10.1007/164_2019_236 31230143

[B29] Song P. Zhang Y. Klaassen C. D. (2011). Dose-response of five bile acids on serum and liver bile acid concentrations and hepatotoxicity in mice. Toxicol. Sci. 123, 359–367. 10.1093/toxsci/kfr177 21747115 PMC3179674

[B30] Sunny N. Parks E. Browning J. (2011). Excessive hepatic mitochondrial TCA cycle and gluconeogenesis in humans with nonalcoholic fatty liver disease. Cell Metab. 14, 804–810. 10.1016/j.cmet.2011.11.004 22152305 PMC3658280

[B31] Takahashi S. Fukami T. Masuo Y. Brocker C. N. Xie C. Krausz K. W. (2016). Cyp2c70 is responsible for the species difference in bile acid metabolism between mice and humans. J. Lipid. Res. 57, 2130–2137. 10.1194/jlr.M071183 27638959 PMC5321228

[B32] Tang X. Yang Q. Yang F. Gong J. Han H. Yang L. (2016). Target profiling analyses of bile acids in the evaluation of hepatoprotective effect of gentiopicroside on ANIT-induced cholestatic liver injury in mice. J. Ethnopharmacol. 194, 63–71. 10.1016/j.jep.2016.08.049 27582267

[B33] Theodoridis G. A. Gika H. G. Want E. J. (2012). Liquid chromatography-mass spectrometry based global metabolite profiling: a review. Anal. Chim. Acta 711, 7–16. 10.1016/j.aca.2011.09.042 22152789

[B34] Tian J. Zhu J. Yi Y. Li C. Zhang Y. Zhao Y. (2017). Dose-related liver injury of geniposide associated with the alteration in bile acid synthesis and transportation. Sci. Rep. 7, 8938. 10.1038/s41598-017-09131-2 28827769 PMC5566417

[B35] Wang X. Wang F. Lu Z. Jin X. Zhang Y. (2018). Semi-quantitative profiling of bile acids in serum and liver reveals the dosage-related effects of dexamethasone on bile acid metabolism in mice. J. Chromatogr. B. Analyt. Technol. Biomed. Life. Sci. 1095, 65–74. 10.1016/j.jchromb.2018.07.021 30055378

[B36] Wei Y. Liu M. Liu J. Li H. (2019). Influence factors on the hepatotoxicity of Polygoni Multiflori Radix. Evid. Based. Complement. Alternat. Med. 2019, 5482896. 10.1155/2019/5482896 31662776 PMC6778938

[B37] Woolbright B. L. Li F. Xie Y. Farhood A. Fickert P. Trauner M. (2014). Lithocholic acid feeding results in direct hepato-toxicity independent of neutrophil function in mice. Toxicol. Lett. 228, 56–66. 10.1016/j.toxlet.2014.04.001 24742700 PMC4057375

[B38] Wu C. Chen C. H. Chen H. C. Liang H. J. Chen S. T. Lin W. Y. (2017). Nuclear magnetic resonance-and mass spectrometry-based metabolomics to study maleic acid toxicity from repeated dose exposure in rats. J. Appl. Toxicol. 37, 1493–1506. 10.1002/jat.3500 28691739

[B39] Wu D. Q. Qin R. L. Xu S. F. Xu Y. S. Lu Y. L. Lu Y. F. (2018). Role of Nrf2 pathway on hepatic fibrosis induced by oleanolic acid. J. Zunyi. Med. Univ. 41, 249–255. 10.14169/j.cnki.zunyixuebao.2018.0051

[B40] Yang T. Shu T. Liu G. Mei H. Zhu X. Huang X. (2017). Quantitative profiling of 19 bile acids in rat plasma, liver, bile and different intestinal section contents to investigate bile acid homeostasis and the application of temporal variation of endogenous bile acids. J. Steroid. Biochem. Mol. Biol. 172, 69–78. 10.1016/j.jsbmb.2017.05.015 28583875

[B41] Yu S. Liu H. Li K. Qin Z. Qin X. Zhu P. (2019). Rapid characterization of the absorbed constituents in rat serum after oral administration and action mechanism of Naozhenning granule using LC-MS and network pharmacology. J. Pharm. Biomed. Anal. 166, 281–290. 10.1016/j.jpba.2019.01.020 30682694

[B42] Zhang Y. Li H. Hu T. Li H. Jin G. Zhang Y. (2018). Metabonomic profiling in study hepatoprotective effect of polysaccharides from Flammulina velutipes on carbon tetrachloride-induced acute liver injury rats using GC-MS. Int. J. Biol. Macromol. 110, 285–293. 10.1016/j.ijbiomac.2017.12.149 29305217

[B43] Zhao D. S. Jiang L. L. Fan Y. X. Wang L. L. Li Z. Q. Shi W. (2017). Investigation of Dioscorea bulbifera Rhizome-induced hepatotoxicity in rats by a multi-sample integrated metabolomics approach. Chem. Res. Toxicol. 30, 1865–1873. 10.1021/acs.chemrestox.7b00176 28899093

[B44] Zhao J. Xie C. Mu X. Krausz K. W. Patel D. P. Shi X. (2018). Metabolic alterations in triptolide-induced acute hepatotoxicity. Biomed. Chromatogr. 32, e4299. 10.1002/bmc.4299 29799631 PMC6150827

[B45] Ziberna L. Šamec D. Mocan A. Nabavi S. F. Bishayee A. Farooqi A. A. (2017). Oleanolic acid alters multiple cell signaling pathways: implication in cancer prevention and therapy. Int. J. Mol. Sci. 18, 643. 10.3390/ijms18030643 PMC5372655 28300756

