# Supplementary figures and images for: LC-MS-Based Metabolomic Study of Oleanolic Acid-Induced Hepatotoxicity in Mice
Source: Front Pharmacol. 2020 May 26;11:747. doi: 10.3389/fphar.2020.00747 (PMC7326119; doi:10.3389/fphar.2020.00747)

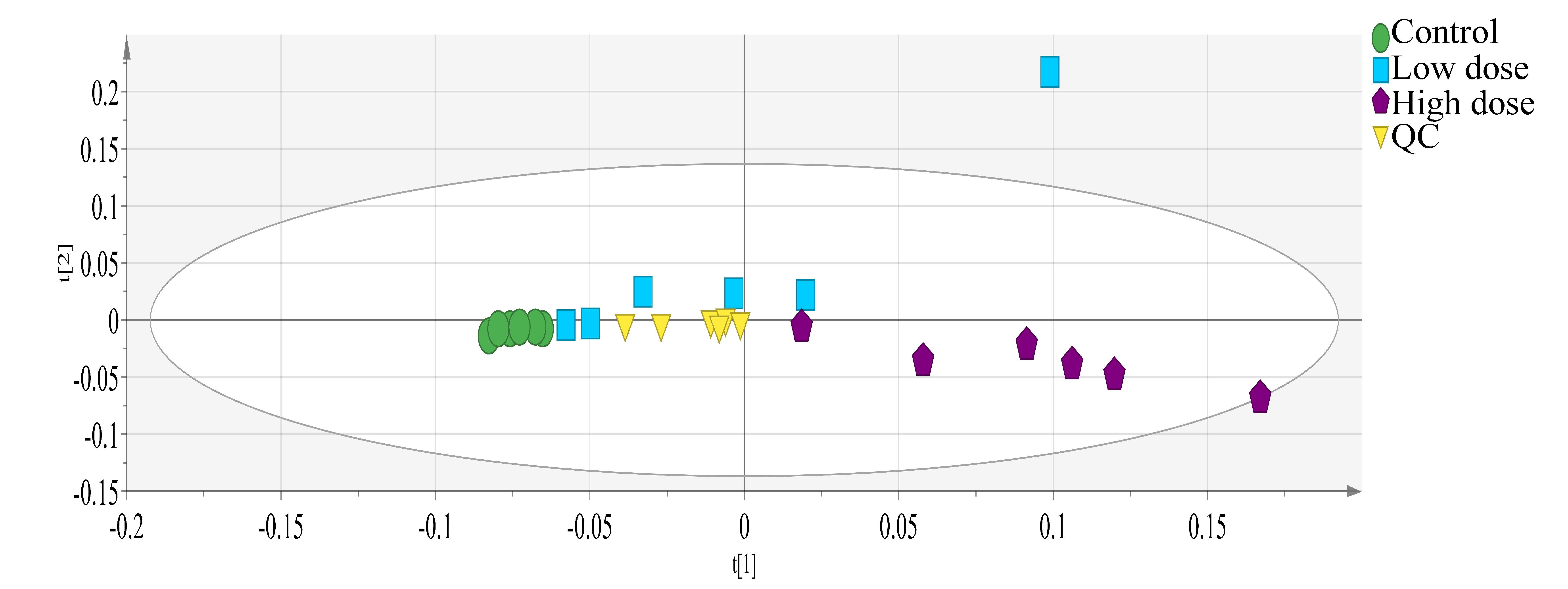

Supplement: Figure S1 — PCA score plots of C57BL/6J mice liver from different groups (n = 6, R2X = 0.593, Q2 543 = 0.217). [file Image_1.tif]

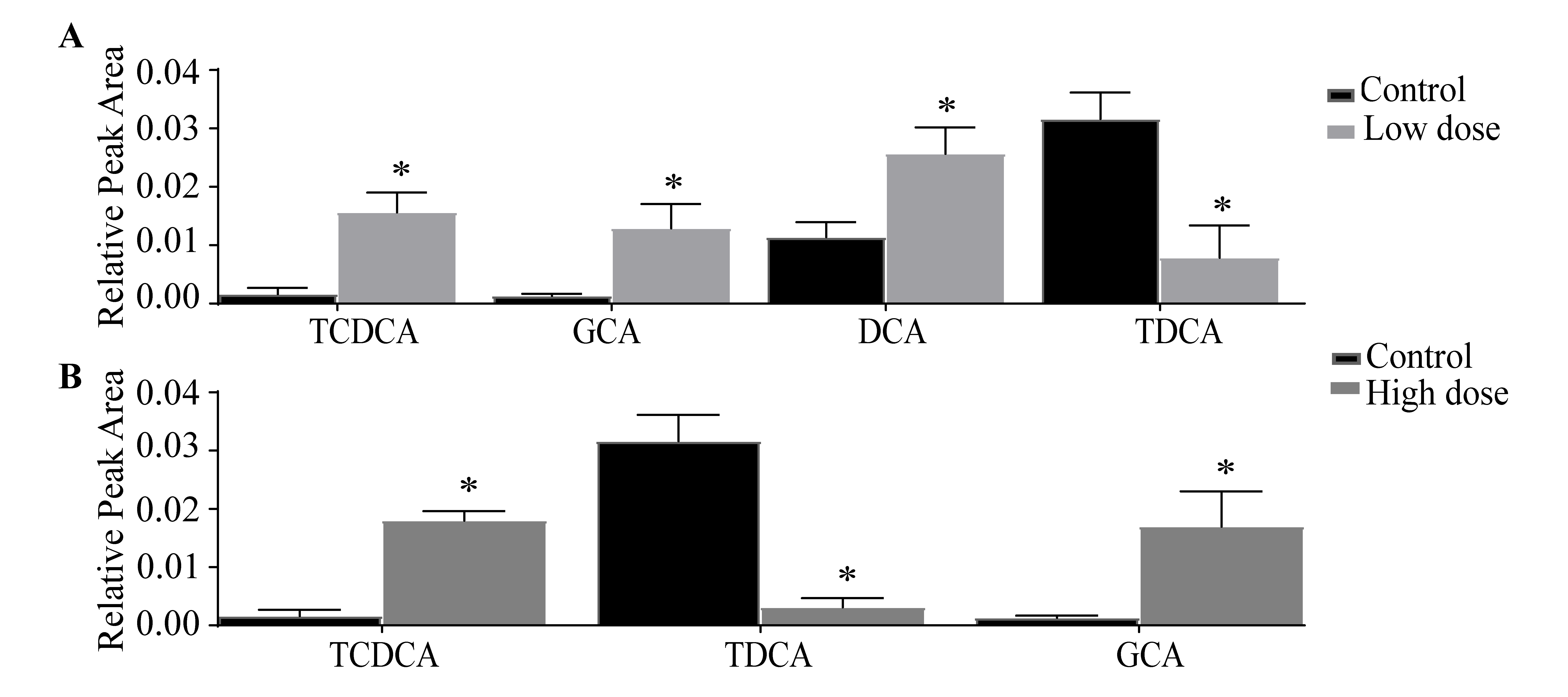

Supplement: Figure S2 — Detailed relative changes in the relative peak area of potential biomarkers. (A) Control group vs low-dose group, (B) Control group vs high-dose group. Data are expressed as mean ± SEM (n = 6). *P < 0.05 vs the control group. [file Image_2.tif]

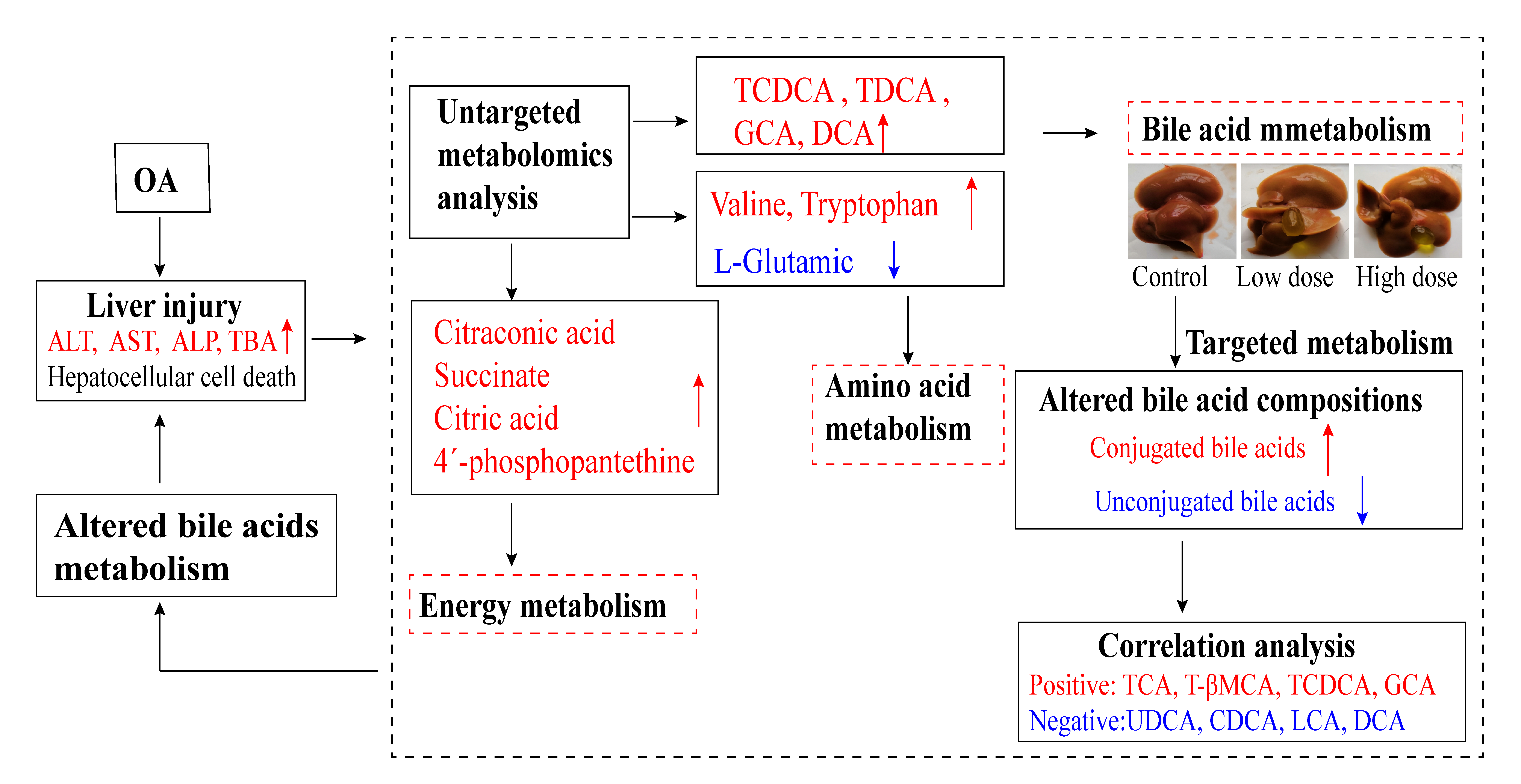

Supplement: Figure S3 — Schematic diagram of OA hepatotoxicity. [file Image_3.tif]
